# When we talk about Big Data, What do we really mean? Toward a more precise definition of Big Data

**DOI:** 10.3389/fdata.2024.1441869

**Published:** 2024-09-10

**Authors:** Xiaoyao Han, Oskar Josef Gstrein, Vasilios Andrikopoulos

**Affiliations:** ^1^Department of Governance and Innovation, Campus Fryslan, University of Groningen, Leeuwarden, Netherlands; ^2^Bernoulli Institute for Mathematics, Computer Science and Artificial Intelligence, University of Groningen, Groningen, Netherlands

**Keywords:** Big Data definition, systematic literature review, scientific research, Big Data review, Big Data epistemology

## Abstract

Despite the lack of consensus on an official definition of Big Data, research and studies have continued to progress based on this “no consensus” stance over the years. However, the lack of a clear definition and scope for Big Data results in scientific research and communication lacking a common ground. Even with the popular “V” characteristics, Big Data remains elusive. The term is broad and is used differently in research, often referring to entirely different concepts, which is rarely stated explicitly in papers. While many studies and reviews attempt to draw a comprehensive understanding of Big Data, there has been little systematic research on the position and practical implications of the term Big Data in research environments. To address this gap, this paper presents a Systematic Literature Review (SLR) on secondary studies to provide a comprehensive overview of how Big Data is used and understood across different scientific domains. Our objective was to monitor the application of the Big Data concept in science, identify which technologies are prevalent in which fields, and investigate the discrepancies between the theoretical understanding and practical usage of the term. Our study found that various Big Data technologies are being used in different scientific fields, including machine learning algorithms, distributed computing frameworks, and other tools. These manifestations of Big Data can be classified into four major categories: abstract concepts, large datasets, machine learning techniques, and the Big Data ecosystem. This study revealed that despite the general agreement on the “V” characteristics, researchers in different scientific fields have varied implicit understandings of Big Data. These implicit understandings significantly influence the content and discussions of studies involving Big Data, although they are often not explicitly stated. We call for a clearer articulation of the meaning of Big Data in research to facilitate smoother scientific communication.

## 1 Introduction

As the amount of data being generated continues to grow over the last two decades (Petroc, 2023), the need to process and analyze it became increasingly urgent. This led to the development of new tools and techniques for handling Big Data (Khalid and Yousaf, 2021), such as distributed computing frameworks like Hadoop and Apache Spark, as well as machine learning algorithms like neural networks and decision trees. As researchers and practitioners in various fields began to integrate Big Data into their work, a body of literature emerged discussing its implementation, characteristics and features, as well as its potential benefits and limitations.

The concept of Big Data has become increasingly important for scientific research. Its characteristics have been extensively discussed in the literature, and numerous researchers have summarized the definitions of Big Data and its closely related features (Khan et al., 2014; Chebbi et al., 2015). Epistemological discussions are common (Kitchin, 2014; Ekbia et al., 2015; Succi and Coveney, 2019) and the recent emergence of new quantitative-based approaches, utilizing large text corpora,offers new opportunities to understand Big Data from different perspectives. Hansmann and Niemeyer (2014), for example, apply a text mining approach to extract and characterize the elements of the Big Data concept by analyzing 248 publications relevant to Big Data. They focus on the concept of the term Big Data and summarize four dimensions to describe it: the dimensions of data, IT infrastructure, applied methods, and an applications perspective. Van Altena et al. (2016), analyze a large number of articles from the biomedical literature to give a better understanding of the term Big Data by extracting relevant topics through topic modeling. Akoka et al. (2017) review studies using a systematic mapping approach based on more than a decade of academic papers relevant to Big Data. Their bibliometric review of Big Data publications shows that the research community has made a significant contribution in the field of Big Data, which is further evidenced by the continued increase in the number of scientific publications dealing with the concept.

Despite the lack of consensus on an official definition of Big Data, research and studies have continued to progress based on this “no consensus” stance over the years. Many authors endeavor to provide comprehensive definitions of Big Data based on their research, aiming to better capture its essence. However, a universally accepted definition is yet to emerge. Instead, a commonly accepted description portrays Big Data through various “V” characteristics (volume, variety, velocity, etc.). Nonetheless, this widely accepted description does not ensure a profound common ground for discussing Big Data in different contexts. As a result, Big Data is still being perceived as a broad and vague concept, making it difficult to grasp in interdisciplinary contexts. Over the past few decades, there has been little systematic research on the position and practical implications of the term Big Data in research environments. When different authors spend significant portions of their studies discussing similar definitions and characteristics of Big Data, they are actually referring to different concepts (technology, platforms, or datasets), which is often not explicitly stated. Exploring this ambiguity is crucial, as it forms the basis for research and discussion. Many reviews aim to enrich a comprehensive understanding of Big Data, rather than retrospectively observing its actual usage in different contexts. we believe this inspection on the current useage sitation of Big Data will help to clarify the ambiguities and facilitates clearer communication among researchers by providing a understanding framework of what Big Data entails in different contexts.

To address this gap, this paper presents a Systematic Literature (SLR) on secondary studies (Kitchenham et al., 2009, 2010) to provide a comprehensive overview of how Big Data is used and understood across different scientific domains. Our objective was to monitor the application of the Big Data concept in science, identify which technologies are prevalent in which fields, and investigate the discrepancies between the theoretical understanding and practical usage of the term.

The rest of this paper is structured as follows: we first present our systematic literature review methodology. The results are then divided into four sections: an overview of the bibliographic information of the extracted articles, a summary of the most prominent technologies used, a discussion on the technologies that are considered to be Big Data, and a presentation of the perceived benefits and drawbacks of Big Data. Finally, before conclusion we discuss our findings in terms of the understanding of Big Data and its value, and suggest avenues for further research.

## 2 Methodology

This study employs a SLR methodology to investigate the use of Big Data in scientific research. Our review, which follows the guidelines for SLR defined by Kitchenham et al. (2009) and Kitchenham et al. (2010), enables a structured and replicable procedure that increases the reliability of research findings. An SLR study is referred to as a tertiary study when it applies the SLR methodology on secondary studies, i.e. it surveys literature reviews instead of primary studies. We selected a tertiary approach for this study due to the extensive range of Big Data technologies and applications in research. Collecting primary data from a large number of papers across domains can be a laborious task. By analyzing secondary data sources instead, our approach offers a comprehensive and holistic overview of the landscape of Big Data in the scientific community. Adopting this approach allows us to better control the bibliographic selection and obtain a comprehensive overview of the field, thereby ensuring high-quality data analysis.

The research questions addressed by this paper are:

1. Which technologies are considered as Big Data in different fields?
2. What Big Data implies in a scientific study context?
3. What is the perceived impact of the adoption of Big Data in each research domain?

For the purpose of answering RQ1, we have referenced the Big Data technology taxonomy developed by Patgiri (2019). We anticipated that the reviewed papers would mention a broad spectrum of technologies, techniques, applications, and heterogeneities, which could pose a challenge in synthesizing the results. Therefore, we relied on Patgiri's taxonomy to guide our full-text review and data extraction process to identify the Big Data technologies utilized in the various scientific domains. This taxonomy offers a comprehensive overview of the various technical approaches to Big Data. By utilizing this taxonomy, we aim to initiate a discourse on what Big Data truly represents in each research domain, and how it has been understood by the respective scientific community. This discussion will enable us to further deliberate on RQ2, which examines the perception of Big Data in specific research domains. To structure our investigation for both research questions (RQ1 and RQ2) we utilize the subject taxonomy of Web of Science (WoS) to classify scientific fields, and apply no limitations to the scope of included scientific fields. As a result, both questions encompass a wide range of Big Data usage.

RQ2 is rooted in the fact that the scope of Big Data technologies has yet to be strictly defined and reach a consensus. Through this question we aim to investigate which technologies or objects are perceived as constituting Big Data in specific research domains. By these means we attempt to shed light on how Big Data as a concept is implicitly understood in each domain by linking it to specific technologies. In addition to offering an overview of the technological landscape, RQ3 aims to delve deeper into the discussion of the impact of Big Data in each domain by examining the benefits and drawbacks of its adoption, as reported by the examined secondary studies. By doing so, we seek to gain a comprehensive understanding of the role of Big Data in scientific domains.

### 2.1 Study design

This study uses the framework of Population Intervention Comparison Outcome and Context (PICOC) (Petticrew and Roberts, 2008) to guide the research questions and the systematic literature review process, with the aim of exploring the use of Big Data technologies across various scientific domains and understanding the perceived meaning and value of Big Data in the scientific community. The use of the PICOC standard allowed for a structured approach to conducting the study and ensured that all relevant aspects were considered in the data collection, refinement, extraction, and analysis processes (Mengist et al., 2020). The Population of interest consists of studies that explore the state of the art of Big Data in different research domains. Intervention refers to systematic secondary studies, such as systematic literature reviews, mapping studies, and surveys. The Comparison in this study is between the perception of Big Data as a concept and the adoption of related technologies. The desired Outcome of this study is a conceptual mapping of how the term Big Data applied in research across domains. In terms of Context, the study focuses on research domains that use the term Big Data to explore the potential of its technology and tool in advancing research without any restriction on the type of domains.

**Figure 1:**
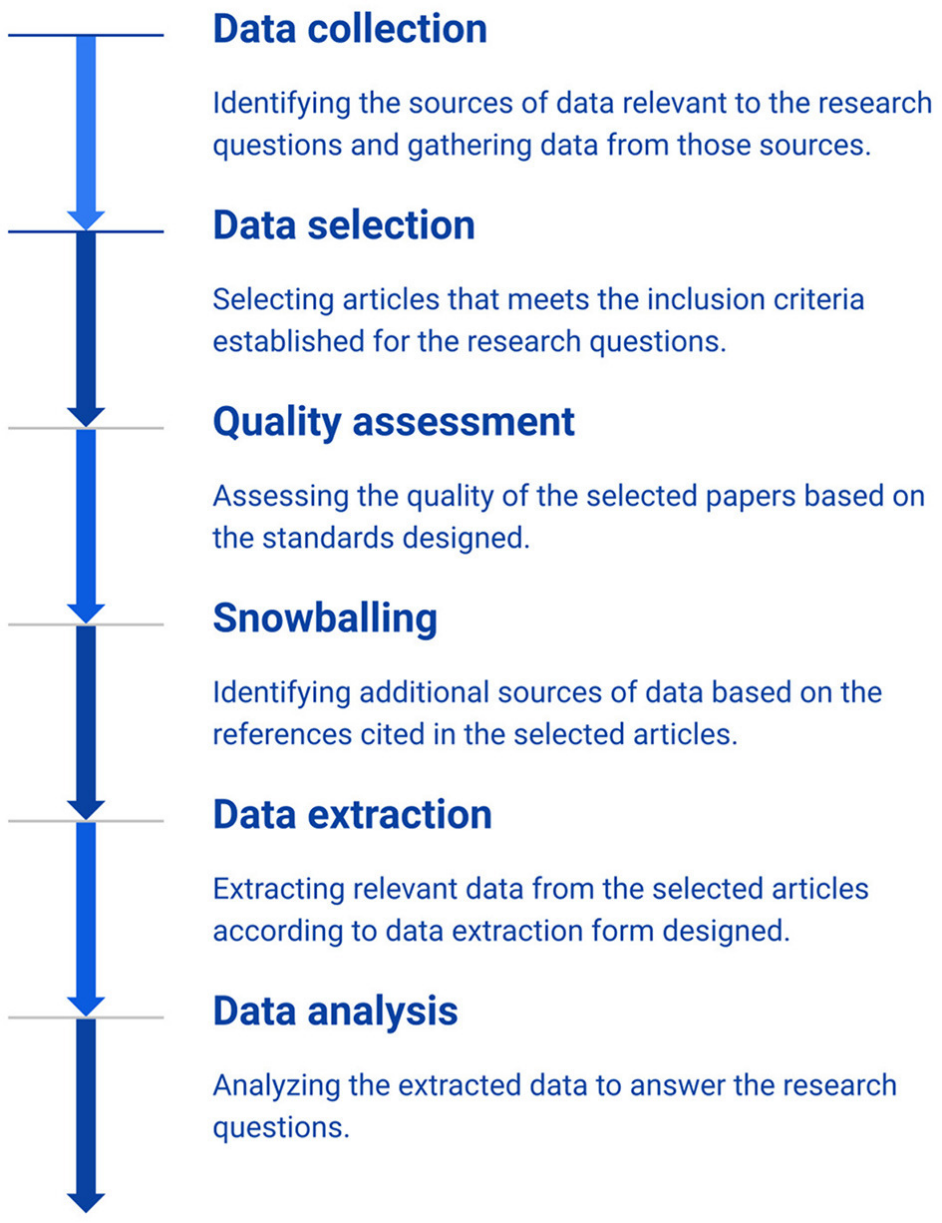
Process of the research methodology.

Figure 1 summarizes the process followed by this SLR. Each of the steps in this process is discussed in more detail in the following.

### 2.2 Data collection

Our aim for data collection was to explore as wide a range of studies in as many different domains as possible. As a result, we decided to retrieve candidate studies through queries on the online databases of Elsevier Scopus, Web of Science, and PubMed. Scopus is a database containing 77 million records from almost 5,000 international publishers (Scopus Content Coverage Guide, 2023), Web of Science is one of the world's leading databases which covers all domains of science (Cumbley and Church, 2013), and PubMed is a comprehensive medicine and biomedical database (Falagas et al., 2007). Our search strategy for all three databases after a few stages of pilot searches, was to look for publications with the exact phrase “big data” and either technology, platform, application, or adoption in the title, along with review, survey, or “mapping study” in either title or keywords.

**Figure 2:**
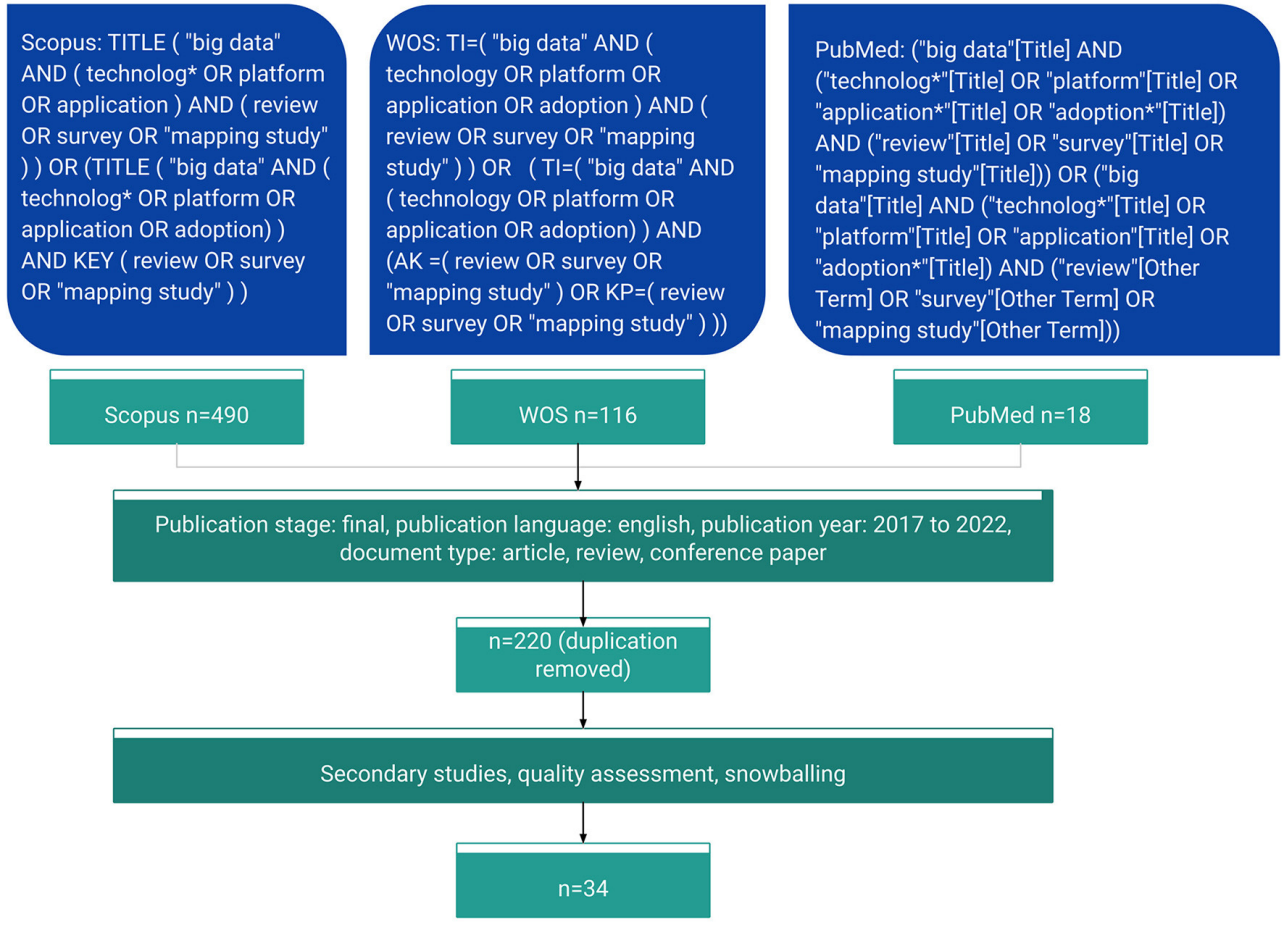
Search queries and screening process.

The specific search query for each database is shown in Figure 2. As the databases use slightly different tags to identify search fields, the search query expression varied but had the same conceptual content. For example, Scopus used the tag TITLE for the title field, whereas WoS used TI and PubMed used square brackets behind the term. For the keywords field, Scopus automatically combined author keywords, index terms, trade names, and chemical names in the tag KEY. WoS provided two relevant fields for keywords, AK representing author keywords and KP representing keywords derived from the titles of article references based on a special algorithm. PubMed placed author keywords and other terms indicating major concepts in the field Other Term. We obtained 490 results from Scopus, 116 from WoS, and 18 from PubMed, resulting in a total of 624 articles before removing duplicates.

### 2.3 Data selection

We established inclusion and exclusion criteria listed in Table 1 to ensure the relevance of the retrieved studies to be included in our analysis. In terms of inclusion criteria, we only considered articles that were published in English between 2017 and 2022 (in order to ensure that the studies reflect the current state of the art in each field), and that were secondary studies. Specifically, we focused on comprehensive reviews or systematic literature reviews that provided an overview of Big Data adoption in a particular research field, rather than articles that focused solely on the application of Big Data technologies to address individual research questions or on the improvement of Big Data technologies themselves. Moreover, we only included articles that were published in their entirety in journals, rather than conference proceedings or abstracts. In contrast, we excluded early access and not final stage articles to avoid potential bias or incomplete information. Publications in languages other than English were also excluded due to language barriers. Additionally, we excluded primary studies, as our aim was to focus on the adoption, application, and implications of Big Data technologies in specific scientific domains, rather than specific research questions.

**Table 1:** Inclusion and exclusion for data selection.

**Inclusion criteria**	**Exclusion criteria**
Publication: finalized	Early access and not final stage articles
Language: english	Languages other than english
Publish time: 2015–2022	Publications before 2015
Secondary studies	Primary studies

By implementing these criteria, we were able to ensure that the articles selected for our analysis were recent, relevant, and provided a comprehensive view of the Big Data landscape. Moreover, by focusing on secondary studies, we were able to avoid redundant coverage of primary research studies and instead obtain a broader perspective on the field. Ultimately, this approach allowed us to generate a more comprehensive and robust understanding of the different Big Data technologies, applications, and techniques being used in various scientific domains. This step resulted in a corpus of 76 records after removing the duplicates.

### 2.4 Quality assessment

To ensure that the papers selected for full-text review meet the requirements of our study, we then conducted a Quality Assessment (QA) based on five standards:

1. Is Big Data adoption discussed at sufficient depth?
2. Is a specific methodology being used in the secondary study?
3. Are the bibliographic sources used included in the study design?
4. Is the number of included primary studies clear in the study?
5. Are the results well-organized?

Firstly, we examined whether the papers provide an overview of Big Data adoption in a particular research field, excluding other aspects such as the application of Big Data technologies that focus on individual research questions or the improvement of Big Data Technologies themselves. Secondly, we assessed whether the selected papers clearly define their research methodology or base it on a specific paradigm. Thirdly, we evaluated whether the papers clearly specify their bibliographic sources for the secondary studies. Fourthly, we documented whether the number of primary papers that are contained in the secondary studies is also clearly stated. Finally, we evaluated whether the results of the papers are well-organized around research questions.

We scored the papers based on these criteria in a binary fashion (fulfilled/not fulfilled), and to be considered for inclusion in our study, we set a threshold of at least three out of five standards that must be fulfilled. While QA is not meant in principle to be used for an additional filtering, we decided that given the observed wide disparities in the quality of the collected secondary studies, setting this threshold is justified. The resulting selected articles primarily use systematic literature reviews, although some pure review articles that are relevant to our study have also been included. This step leads to 33 studies left for further processing.

### 2.5 Snowballing

In addition to the papers collected, we employed the forward snowball sampling method (Wohlin, 2014) to supplement our database. For each article under full-text review passing the QA step, we reviewed all the titles of its references and the articles citing it. We added records that met our inclusion and exclusion criteria and were relevant to our topics. As we are only interested in recent studies on the topic, we did not perform backward snowballing. Ultimately, we selected only one additional study for inclusion, resulting in 34 studies in total for data extraction. All authors actively contributed to each step of the research process, engaging in thorough discussions and collaborations. In every stage outlined above, one researcher took the lead and collaborated with the other two authors to ensure comprehensive coverage and rigorous analysis.

### 2.6 Data extraction

The data extraction form shown in Table 2 was designed to answer the research questions in this study. The extraction fields can be divided into three categories. The first part concerns demographics-related data to be extracted from each study in order to check for possible bias toward specific fields or publication venues. The second part is used to answer RQ1 and RQ2. For the Research Field aspect, we applied the subject classification from WoS to ensure consistency and heterogeneity of the level of the discipline described. This also enabled us to make cross-sectional comparisons and statistical summaries. Apart from the WoS categories, we added another field to specify the sub-level of the Application Domain in which the secondary study takes place. This helps us to better connect the technologies collected with their intended use cases. In terms of the technologies and applications that we collected, due to the wide range of information described from different perspectives and heterogeneity issues, we added one more column to clarify and support the documentation, namely Perspective. This column records the perspective from which the Big Data technologies are described. For example, some papers only summarized the use cases of machine learning methods as applications of Big Data. In this way, besides the concrete names of methods and models documented in the Technologies column (including also specific Big Data applications or platforms), the perspective will be marked as machine learning. This helps us to further analyze and categorize the discussed technologies. Reported shortcomings and benefits of the perceived impact of Big Data from the authors are extracted in the third part. This helps us to answer RQ3.

**Table 2:** Fields of data extraction form.

	**Data extraction fields**	**Description**
Demographics	ID	Number as article identification
	Title	Title of article
	Journal	Journal name
	Publisher	Publisher name
	Year	Publication year
RQ1&RQ2	Research field	Subject according to WoS classification
	Application domains	Sub-level of research field
	Technologies	Specific technologies, applications, or platforms named by the study
	Perspective	Further description of technologies
RQ3	Shortcomings	Text extracted regarding the shortcomings of Big Data discussed in the paper
	Benefits	Text extracted regarding the benefits of Big Data discussed in the paper

### 2.7 Threats to validity

The present study faces several potential threats to its validity. Firstly, the inclusion and exclusion criteria employed could introduce bias into the sample. If the criteria are too narrow, relevant articles may be omitted, while if they are too broad, irrelevant articles may be included, resulting in a less representative sample. To mitigate this threat, we thoroughly reviewed and refined our criteria through piloting to ensure their optimal balance between inclusivity and exclusivity. Secondly, the quality assessment of the chosen articles could be influenced by researcher bias, as different researchers might interpret the quality criteria differently. To address this issue, we utilized a standardized quality assessment tool during the evaluation process to ensure consistent interpretation of the criteria. Moreover, limiting the search to a specific publication date range could result in publication date bias, leading to an outdated or incomplete synthesis of the literature. To avoid this, we focused on secondary studies and restricted the publication date to the latest 5 years, which enabled us to include publications from a wide range of dates. To enhance the efficiency of the quality assessment, we established the quality assessment process before conducting the full-text review. This approach ensured that all selected articles were relevant to the topic and that their quality was assessed rigorously. Other possible threats to the validity of this study include publication bias and selective reporting of results. To mitigate these threats, we conducted a comprehensive search of multiple databases and employed the snowballing method, while carefully screening all articles for possible selective reporting.

## 3 Results

This section presents the results of our study and is divided into several subsections. Firstly, we provide an overview of the background information of the articles in our corpus, including the distribution of disciplines, publishers, and popular databases cited for their secondary studies. The presentation of the results is using the classification of the secondary studies into research domains extracted for RQ1 and RQ2. In Section 3.2, we discuss our findings on the Big Data technologies extracted from our SLR in order to answer RQ1. In Section 3.3, we discuss the concepts that are perceived as Big Data as reported by the secondary studies in our corpus. Finally, we summarize the impact of the adoption of Big Data technologies in the various domains in Section 3.4.

### 3.1 Overview of bibliographic results

The following section presents an overview of the created corpus used in the analysis, providing statistics on domain distribution, publishers, and bibliographic sources. Domain distribution helps to identify the research background of the analyzed articles, while publishers' distribution sheds light on those who are active in the field of Big Data. Additionally, the bibliographic sources used by secondary studies provide insights into the popularity of different databases and sources among researchers in the field. This information is crucial since it can affect the quality of the data and the generalizability of the findings and ensure transparency for analysis.

**Figure 3:**
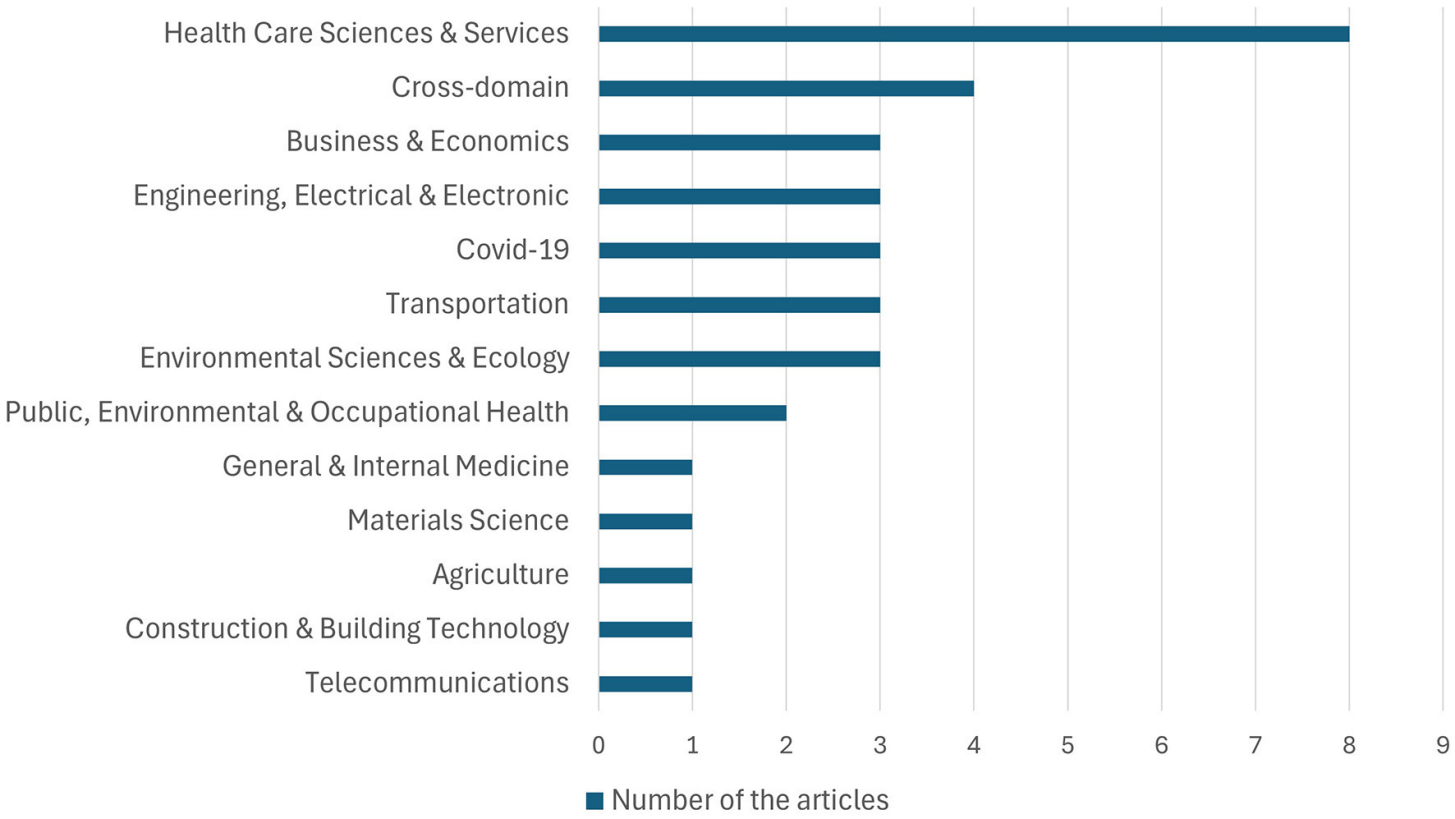
Publisher distribution of included articles.

Figure 3 shows the distribution of WoS-defined disciplines of the articles extracted for the study. Health care emerged as the discipline that includes the most Big Data articles, with COVID-19 as a distinct area of interest that we promoted to its own domain. Transportation and Business & Economics were the next most popular areas of study. While several cross-domain studies did not specify the application domain of Big Data, they did focus on a specific aspect of Big Data applications such as storage and cybersecurity. Disaster response studies identified in the Public, Environmental & Occupational Health category are also notable. Other disciplines appear only once; these include Material Science that focused on Aerospace, Telecommunications (e.g. mobile Big Data), Construction & Building Technology, and Agriculture. It should be noted that this distribution does not necessarily represent the entire scientific community, and no conclusions regarding the interest of studying or using Big Data can be drawn from this.

**Figure 4:**
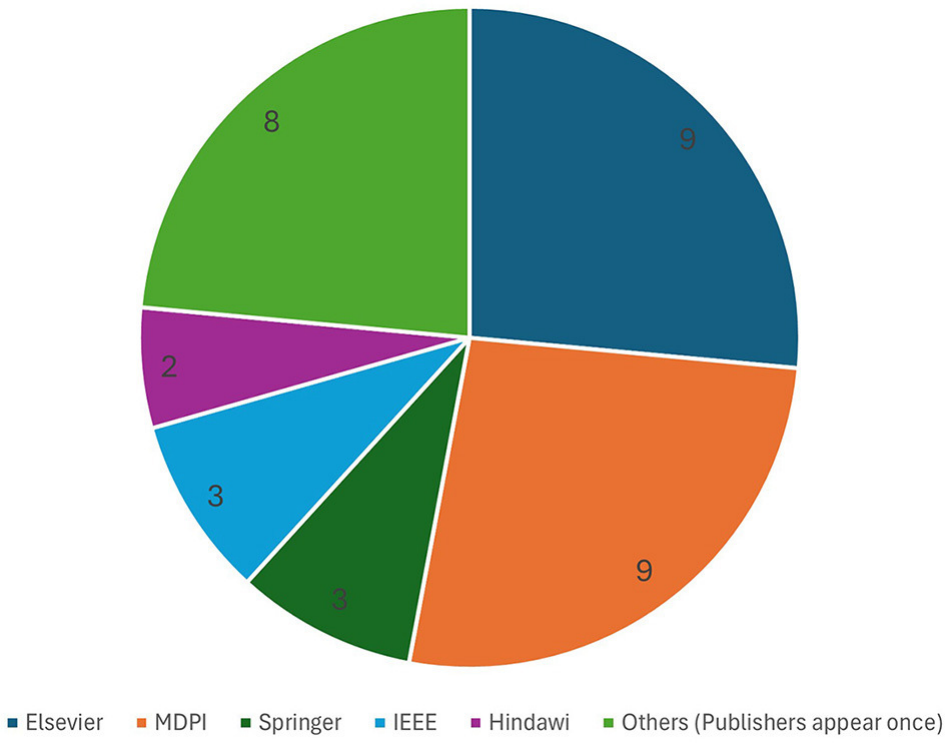
Publishers' distribution of the included secondary studies.

Our analysis of the articles in our corpus, as summarized in Figure 4, showed that MDPI and Elsevier published the most studies related to Big Data (with nine each), followed by Springer and IEEE (with three each). Eight more publishers have one publication each in our corpus and are omitted from the figure.

**Figure 5:**
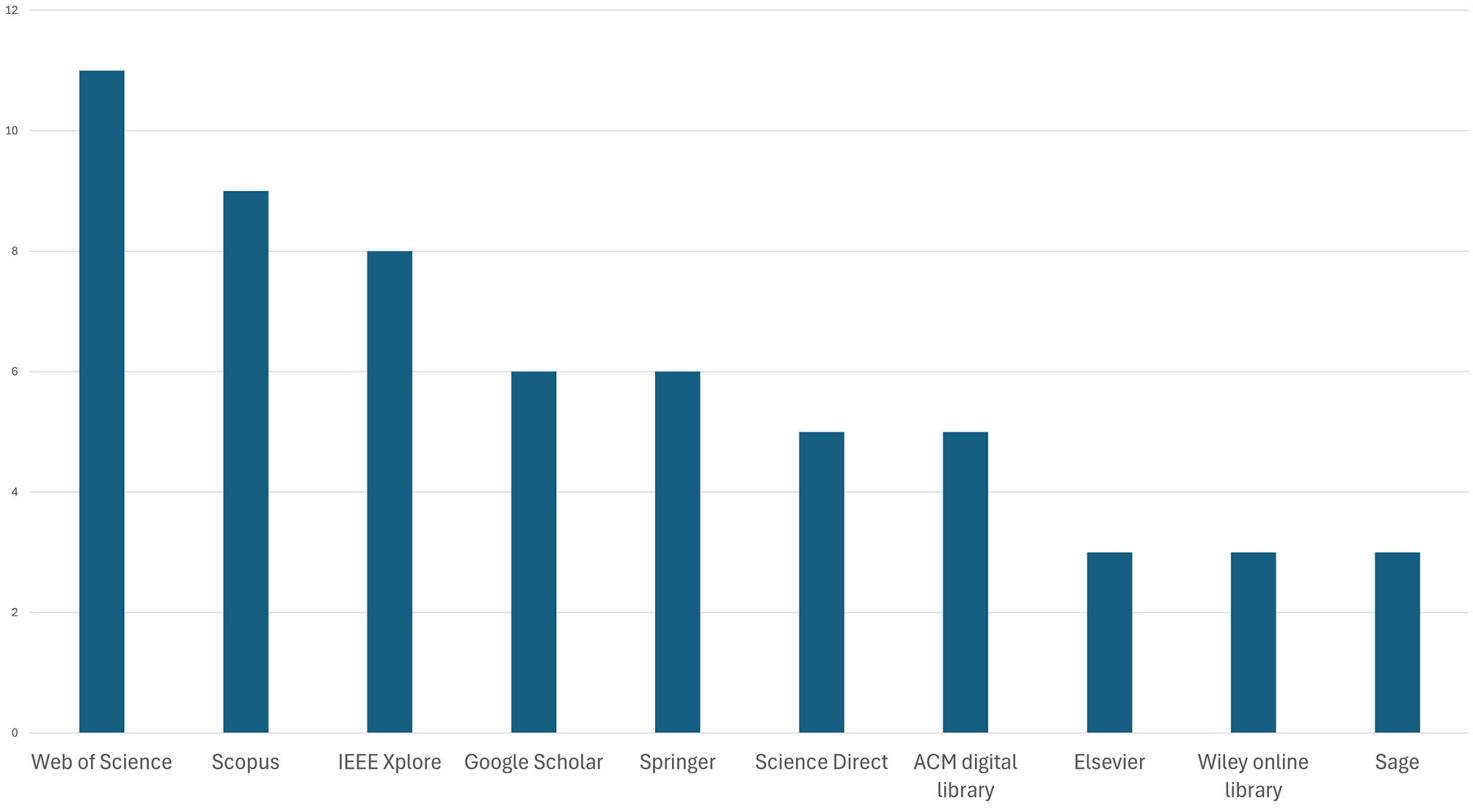
Popularity of databases used by the secondary studies.

In terms of databases chosen for secondary studies, Figure 5 shows that WoS and Scopus were the most popular sources, being in alignment with our choice of databases for our study, followed by IEEE, ScienceDirect, Google Scholar, Wiley Online Library, Sage, PubMed, and Association for Computing Machinery's (ACM) Digital Library.

Overall, a wide diversity of publishing venues and data sources can be attested for our corpus, providing evidence toward a lack of selection bias for our review.

### 3.2 Big Data technologies

To address RQ1, we refer to Patgiri (2019), who presents a taxonomy of Big Data consisting of seven key categories: Semantics, Compute Infrastructure, Storage System, Big Data Management, Big Data Mining, Big Machine Learning, and Security & Privacy. This taxonomy covers various aspects of Big Data technologies, including implementation tools, system architectures, and operational processes. We categorize and describe our findings based on the six perspectives (excluding Semantics) proposed by Patgiri. Since the Semantics perspective primarily concerns the conceptual understanding of Big Data, we exclude it from our summary of technologies.

The data extraction results from our study reveal the use of various Big Data technologies already discussed in Patgiri (2019), including Xplenty, Apache Cassandra, MongoDB, Hadoop, Datawrapper, Rapidminer, Tableau, KNIME, Storm, Cloudera Distributed Hadoop(CDH), Kafka, Spark, Mapreduce, Hive, Pig, Flume, Sqoop, Apache Tez, Flink, and Storm. These technologies are used to extract, process, analyze, and visualize large volumes of data across a range of scientific domains.

From the Compute Infrastructure perspective, the most commonly used technology is Hadoop,^1^ which is used for distributed computing and data processing. Various disciplines have been found utilizing Hadoop, including health care [S12, S63, S87, S133], environmental science [S77], and transportation [S194]. Other technologies, such as Apache Cassandra^2^ and MongoDB,^3^ are used for distributed data storage and retrieval. These technologies enable data processing at scale and facilitate parallel processing, enabling users to analyze large volumes of data quickly and efficiently. In terms of storage systems, Apache Cassandra, Hadoop Distributed File System (HDFS),^4^ HBase,^5^ and MongoDB are popular choices for distributed data storage. These technologies are designed to handle large volumes of data and provide high scalability and fault tolerance. The reported application domains for these technologies include Internet of Things (IoT), healthcare, decision making and electric power data [S94]. [S133] notes that NoSQL database systems such as MongoDB, Cassandra, and HDFS can be used to handle exponential data growth to replace the traditional database management systems. Additionally, technologies such as Flume^6^ and Sqoop^7^ enable the efficient transfer of data between different storage systems [S74].

Regarding the Big Data Management perspective, frameworks such as Hadoop, MapReduce, Hive,^8^ and Pig^9^ are used for managing and processing large volumes of data. These technologies enable users to process data in a distributed environment, thereby increasing efficiency and scalability [S74, S133, S194]. Other technologies, such as Apache Tez^10^ and Flink,^11^ provide high-performance data processing and streaming capabilities [S74]. From the Big Data Mining & Machine Learning aspect, a range of machine learning models are used to analyze large volumes of data, including Artificial Neural Network (ANN), fuzzy logic, Decision Tree (DT), regression, Support vector machine (SVM), Self-Organizing Map (SOM), Fuzzy C-Means (FCM), K-means, Genetic algorithm (GA), and Convolution Neural Networks (CNN), DT, Random Forest, Rotation Forest, Classification And Regression Trees, Bayesian, Boosted Regression Tree, (CART), Conditional Inference Tree, Maxent, K-means, Non-negative Matrix Fact, and others [S19, S27, S65, S96, S178, S203, S207]. These techniques enable users to extract insights from large volumes of data, identify patterns and trends, and make predictions based on data analysis. Finally, as for security and privacy, Intrusion Detection Systems (IDS) are used to detect anomalous behaviors in complex environments. Machine learning models such as unsupervised online deep neural networks and deep learning techniques are used to identify and analyze Controller Area Network (CAN) attacks. Log parsers such as Zookeeper,^12^ Proxifier,^13^ BGL, HPC and HDFS are used to secure data and ensure privacy in distributed environments [S88]. These technologies provide access control, authentication, and encryption capabilities to protect data against unauthorized access and misuse.

Although many Big Data technologies and their applications are presented in this section, a significant portion of our research found that there are additional technologies and applications that are discussed outside of Patgiti's taxonomy, yet are still labeled as Big Data. In order to distinguish between these two cases and further deepen the understanding of Big Data and related technologies in the scientific field, we discuss the latter case in the following research question.

### 3.3 Perceptions of Big Data scope

In this section, we aim to provide an overview of the landscape on how Big Data is actually used based on our analysis of the literature to answer RQ2. Initially, we attempted to use the WoS categories to structure the discussion around domains; however, due to the heterogeneity issue at different angles, the difference in technology use was found to be significant even within one subject. As a result, we summarize the technologies according to the Perspective field recorded in the data extraction form. Table 3 summarizes our findings.

**Table 3:** Perceptions of Big Data scope.

**Category**	**Domain**	**Studies**
Articles not mentioning specific technologies	Business & Economics	67
	COVID-19	104
	Cross-domain	181
Big Data as large data sources	Environmental Sciences & Ecology	16
	Health Care Sciences & Services	47, 87
	Engineering, Electrical & Electronic	66
	COVID-19	132
	Cross-domain	181
Machine learning methods and models	General & Internal Medicine	12
	Public, Environmental & Occupational Health	15
	Cross-domain	19, 203
	Transportation	27
	Telecommunications	71
	Engineering, Electrical & Electronic	96
	Construction & Building Technology	135
	Agriculture	178
	Health Care Sciences & Services	207
Big Data ecosystem	Business & Economics	133
		Health Care Sciences & Services	94
		Public, Environmental & Occupational Health	172
		Materials Science	194,216

#### 3.3.1 Big Data as abstract concepts or collections for application domains

In this category, the studies do not exemplify the Big Data technologies in concrete circumstances, but rather as concepts or collections for application domains. For example, [S67] discusses using text mining methods to explore the application of Big Data disciplines. The research focuses on the broad application of Big Data but does not include specific technologies. Based on the presented text mining method, Big Data technologies are identified automatically as keywords without further classification, and no concrete technologies and their use cases are explored. Similarly, [S104] studies the implementation of cloud computing, Big Data, and blockchain technology adoption in Enterprise Resource Planning (ERP), using only publication keywords generated from literature review and covering a broad spectrum. Why certain technologies are being referred to as Big Data, and how they are specifically utilized is missing, however. In another case, the term “Big Data” and related expressions such as “Big Data Analytics” and “Big Data Applications” are used as abstract concepts throughout the paper without a clear definition of the scope of the technology or little mention of the technology itself. For instance, in an attempt to identify privacy issues in Big Data applications, [S181] uses phrases such as “Big Data analytics” to refer to the technology, without focusing on any specific technologies.

#### 3.3.2 Big Data as large data sources

This category of studies focuses on newly established large data sources, such as databases, websites, and crowd-sourcing platforms, which are taken as the application target of Big Data. The term Big Data is not strictly focused on the technologies but rather the volume or velocity of the data, with no specific techniques linked to those data sources. The application of Big Data in this case mainly means the use of large volumes of data. For example, [S132] explores the utilization of Big Data, smart and digital technologies, and artificial intelligence in the field of psoriatic arthritis studies. The authors cite a series of examples of how Big Data, combined with techniques such as artificial neural networks, natural language processing, k-nearest neighbors, and Bayesian models, can help intercept patients with psoriatic arthritis early. In this case, Big Data mainly refers to the repositories, registries, and databases that are generated from surveys, medical insurance data, vital registration data, etc. A similar research study in healthcare is [S47]. The authors list a number of databases worldwide with concrete gastrointestinal and liver diseases sample sizes, and techniques that are briefly mentioned for analysis are R, python, statistics, and Natural Language Processing (NLP). Also in ecology, ecological datasets such as AmeriFlux are considered Big Data as indicated by [S16].

#### 3.3.3 Machine learning methods and models

This category refers to Big Data as models, algorithms, and statistical methods for managing large data sets, closely related to Machine Learning, Artificial Intelligence, Data Mining, Internet of Things, etc. Big Data technology is equivalent to the definition of machine learning or artificial intelligence, sometimes referred to as deep learning. Specific machine learning models are mentioned, such as ANN, Neural Network (NN), SVM, K-means, decision trees, and also improved models that are based on those methods, to solve practical problems, such as prediction for a certain disease, optimization of transportation. In our research, the majority of the technologies and applications extracted fall into this category. Popular models and techniques summarized are decision trees, random forests and support vector logistic regression models, naive Bayes models, decision trees, and k-nearest neighbors (k-NN) classic linear statistic models, Bayesian networks, and CNNs. There are also some studies (e.g. [S203, S207]) that summarize the applications of Big Data from their research field by discussing a series of concrete improvements of existing models.

#### 3.3.4 Big Data ecosystem

The Big Data ecosystem category represents a comprehensive structure of the different levels of technology solutions that exist. Various applications such as Hadoop, MapReduce, and NoSQL are commonly mentioned in this category. The authors of those studies show a strong familiarity with how these technologies fit into their respective disciplines, demonstrating a deep understanding of the subject matter. In one study [S194], for example, the authors explained the different layers in an architecture for Big Data relating to traffic, and how applications such as Hadoop, MapReduce, HDFS, Apache Spark, Apache Hive, Hut, and Apache Kafka work together in the system. The article provides valuable insights into the role that these applications play in the overall architecture. Another study [S216] focused on the use of MapReduce and HDFS functionality in Big Data Architecture.

#### 3.3.5 Outside of the classification

Some articles in our study did not fit into the categories outlined in our classification, as it is challenging to extract useful information from them. For instance, some studies e.g., [S12,S63,S77,S172] presented a comparison of Big Data frameworks sourced from other literature without exploring how these frameworks are utilized in their respective fields after introducing their background discipline. While these frameworks can be categorized into the fourth category, we found it difficult to ascertain how Big Data is applied and understood in these disciplines. As a result, we did not include them in Table 3. Articles that do not fit into this classification do not imply that the classification is invalid for the study, but rather that we cannot accurately estimate the authors' understanding of Big Data.

### 3.4 Added value of Big Data

In this section, we summarize the perceived impact of Big Data adoption to each domain, as stated in the secondary studies and in order to answer RQ3. The impact of Big Data is categorized into benefits and shortcomings, which will be introduced below. Table 4 provides a summary of the main points extracted from the texts.

**Table 4:** Summary of benefits and shortcomings of Big Data.

**Benefits**	**Shortcomings**
Ability to store, process, and analyze large volumes and varieties of data in real-time	Assumption that having vast amounts of data guarantees accurate results
Cost reduction in medical treatments, disease prediction, improved preventative care, and medication efficiency analysis in healthcare	Waste of resources if used unnecessarily
Reliable capture of small variations in incidence or disease flare	Physical challenges to current IT architecture, servers, and software
Improved effectiveness of traffic crash detection and prediction research	Security and privacy issues
Revealing previously overlooked correlations, market trends, and valuable information from a large amount of big data	Challenges in managing data consistency, scalability, and integration in different fields

#### 3.4.1 Benefits

The advantages of Big Data in various fields have been extensively researched and documented. Big Data tools have the ability to store, process and analyze large volumes and varieties of data in real time, enabling researchers to extract valuable insights and improve their performance. In healthcare, the integration of different scientific fields such as informatics, clinical sciences, and analytics has been facilitated by the application of Big Data [S12]. Further reported benefits of Big Data in healthcare include, for example, cost reduction in medical treatments, elimination of illness-related risk factors, disease prediction, and improved preventative care and medication efficiency analysis [S12, S47, S132, S181]. Large sample sizes have enabled reliable capture of small variations in incidence or disease flare, and epidemiological/clinical Big Data has been instrumental in guiding public and global health policies [S47, S132]. In the field of transportation, Big Data technologies have been used to improve the effectiveness of traffic crash detection and prediction research, with the aim of preventing the occurrences of traffic crashes and secondary crashes [S178, S194]. In the ecosystem service research, Big Data and machine learning tools have been used to address data availability, uncertainty, and socio-ecological gaps [S16]. In addition, Big Data analytics platforms have been useful in revealing previously overlooked correlations, market trends, and valuable information from a large amount of Big Data [S66]. Machine learning has also been used in the smart grid area to sift through Big Data and extract useful information that can aid in demand and generation pattern recognition [S51]. Overall, the benefits of Big Data are diverse and far-reaching, and its application has the potential to revolutionize research and improve outcomes in various fields.

#### 3.4.2 Shortcomings

One of the primary challenges with Big Data is the assumption that having vast amounts of data guarantees accurate results. As [S74] notes, Big Data can give a false sense of security because having a lot of data does not necessarily mean that the results are valid. In the field of Psoriatic Arthritis, for example, there are significant variations in socio-demographic characteristics, co-morbidities, and major complication rates between individual (single- or multi-center) and database-based studies [S132]. This inconsistency raises concerns when critically appraising rheumatological and dermatological research, as well as risk adjustment modeling.

Another challenge with Big Data is that unnecessary utilization of Big Data can lead to a waste of resources, as it ties up computer resources [S74]. With the exponential growth of data, the storage and processing demands for Big Data have increased significantly. Unnecessary utilization of Big Data can exhaust computer resources and make them less available for other important tasks. This can result in increased costs for organizations, as they need to invest in more powerful computer systems to handle the increased demand. Big Data also poses physical challenges to current IT architecture, servers, and software. As pointed out by [S51], IoT devices generate a huge amount of data, which cannot be handled through conventional analysis techniques. While many data storage technologies have been proposed to store and process growing data, more efficient technologies are required for data acquisition, processing, pre-processing, storage, and management of Big Data [S94].

Security and privacy issues also arise with Big Data. In the healthcare industry, data security and patient privacy issues are a significant concern for authorities and patients [S66]. In the research concerning COVID-19 [S207], for example, there are ethical issues surrounding privacy, the use of personal data to limit the pandemic spread, and the need for security to protect data from being overused by technology.

Finally, there are challenges in managing data consistency, scalability, and integration. For instance, challenges in using Big Data in environmental Sciences include data cleansing, lack of labeled datasets, mismatched data ingestion, high costs of platforms, and lack of data governance and socio-technical infrastructure [S135].The transportation industry faces difficulties in collecting data from diverse sources and addressing quality concerns. When using Big Data in transportation, data collected from different sources needs to be analyzed to extract meaningful insights. However, this data often contains noise and uncertainty that must be addressed before use [S194].

In conclusion, several challenges need to be addressed to ensure that the use of Big Data is effective. These challenges include the need for integrated and comprehensive systems, efficient data acquisition and management technologies, security and privacy concerns, data consistency, scalability, as well as integration issues.

## 4 Discussion

Going beyond our findings as reported in the previous section, in the following we identify three specific topics emerging from our analysis that need further consideration. These are how Big Data is being understood across scientific domains, how the adoption of the Big Data occurs across disciplines, and how the perception of the added value that Big Data technologies bring looks like.

### 4.1 Understanding of Big Data

The challenge in analyzing research using Big Data technologies stems from the lack of a clear definition of what a Big Data technology is. The authors of the secondary studies surveyed through this study elaborate on their understanding of Big Data and its applications from different perspectives. This results in a wide spectrum of technologies which are labeled as Big Data, while significant differences between them remain, even when applied in the same field. Additionally, terms closely related to Big Data technology, such as AI, ML, Big Data analytics, Big Data platform, IoT, and Deep Learning are often used interchangeably. While these terms may not require definition in each individual article surveyed, their meanings and scope may overlap in practice. Therefore, to gain a clear understanding of Big Data, it would be helpful to have a clear distinction and consistent use of each term with more precision to avoid confusion. In other words, we argue that when reviewing the literature, the primary task is to understand what authors mean when they use concepts such as Big Data and Big Data technology, rather than simply extracting relevant technical applications and their context.

The results show that the concept of Big Data technology can be very broad. At the same time, these tools and technologies may be specific to a particular discipline and may not have interdisciplinary significance. This ambiguity has led to the widespread use of Big Data terminology in some cases. Our findings also revealed that some articles provide a vague description of Big Data, making it difficult to extract useful information that cannot be categorized under any of the categories in Section 3.3. These articles focus on the theoretical knowledge of Big Data and do not delve into its practical applications in different domains. As a result, they provide a pure comparison of Big Data frameworks cited in other literature without exploring how these frameworks are used in their respective domains. This approach contributes to pervasive references to Big Data, however, without providing a clear understanding of its practical applications.

In conclusion, this study highlights the need for a clear and comprehensive definition of Big Data technology and its practical applications in different domains. By doing so, we can avoid the misuse of Big Data terminology and gain a better understanding of the tools and technologies that are truly related to Big Data.

### 4.2 Range of disciplines represented in Big Data applications

In this study, we conducted a rigorous systematic literature review to investigate the applications of Big Data and used the WoS subject classification to categorize the related research fields. However, we acknowledge that this approach may not capture the full range of disciplines, as indicated by our analysis of the discipline distribution. While disciplines such as astronomy and high-energy physics are typically associated with managing large volumes of data (Jacobs, 2009), we found that they were not represented in the papers we extracted. The lack of such data-intensive disciplines led us to question why they were not included, since our primary objective was to provide an overview of Big Data applications across the entire scientific community. We took great care to ensure that our SLR was not biased toward any specific domain by carefully selecting keywords and defining our inclusion and exclusion criteria. Additionally, we used comprehensive databases and an auditable and repeatable methodology. The absence of data-intensive subjects in our review suggests that some disciplines may be utilizing Big Data technologies without explicitly using the term “Big Data” in their research papers. One hypothesis is that computer science and related fields may consider the scale of data used as the norm, hence not explicitly mentioning “Big Data.” To understand this phenomenon better, we suggest a further step to investigate the underlying assumptions, such as the choice of terminologies, used in these disciplines as part of future work.

### 4.3 Value of Big Data

The enthusiasm surrounding Big Data arises from the belief that vast amount of information with the development of technologies, algorithms and machine learning techniques, can provide innovative insights that traditional research methods cannot achieve (Agrawal et al., 2011; Chen and Zhang, 2014; Khalid and Yousaf, 2021). However, these optimistic views are not undisputed. As Big Data knowledge infrastructures emerge, researchers are increasingly discussing the challenges and limitations they present.

Numerous papers extol the merits of Big Data, with the main claim being that it offers unparalleled opportunities for scientific breakthroughs, leading to transformative research, as discussed in Section 3.4. The most significant advantage of the Big Data approach is its ability to address problems on larger and finer temporal and spatial scales, as well as provide information on which data are reliable or uncertain, thereby mapping ignorance (Hampton et al., 2013; Harford, 2014; Isaac et al., 2020). Big Data also highlights blind spots and uncertainties in research, revealing gaps in existing knowledge (Hortal et al., 2015). In this study, we have explored the benefits of Big Data tools. They can store, process, and analyze large volumes and varieties of data in real-time, enabling researchers to extract valuable insights and improve their performance. Big Data analytics platforms have proven useful in revealing previously overlooked correlations, market trends, and valuable information from large datasets. Additionally, machine learning has aided in sifting through Big Data to extract useful information for demand and generation pattern recognition in the smart grid.

However, according to Ekbia et al. (2015), Big Data presents both conceptual and practical dilemmas based on a broad range of literature. They argue that an epistemological shift in science occurs due to the use of Big Data, where predictive modeling and simulation gain more importance than causal explanations based on repeatable experiments testing hypotheses. The authors Rosenheim and Gratton (2017) reject what they perceive as the suggestion of the most fervent proponents of Big Data that knowledge of correlation alone can replace knowledge of causality. They point out that understanding cause-and-effect relationships is critical in fields such as agricultural entomology, where research-oriented recommendations enable farmers to implement management actions that lead to desired outcomes. The study in Harford (2014) points out that conducting a correlation-based analysis without a theoretical framework is inherently vulnerable. Without understanding the underlying factors influencing a correlation, it becomes impossible to anticipate and account for factors that could potentially compromise its validity.

Similarly to the above, in our research, we found critical questions regarding whether vast amounts of data guarantee accurate results. Brady (2019) argues that social scientists must grasp the meaning of concepts and predictions generated by convoluted algorithms, weigh the relative value of prediction vs. causal inference, and cope with ethical challenges as their methods. Another notable challenge posed by Big Data that we found is managing data consistency, scalability and heterogeneity in different fields. Additionally, large amounts of data also pose challenges to IT architecture, servers and software. This was also testified by Fan et al. (2014). They point out that the massive sample size and high dimensionality of Big Data introduce unique computational and statistical challenges, including scalability and storage bottleneck, noise accumulation, spurious correlation, incidental endogeneity and measurement errors. Furthermore, we found that the misuses of Big Data arouse ethical issues. This concern has already been widely discussed in previous literature, see for example Cumbley and Church (2013) and Knoppers and Thorogood (2017).

In conclusion, while Big Data has generated much enthusiasm as a powerful tool for scientific breakthroughs, it is not without its challenges and limitations. Despite these challenges, Big Data holds promise as a tool for innovative research, and future work should continue to address these concerns while exploring new opportunities for knowledge discovery.

## 5 Conclusion

The lack of a clear definition and scope for Big Data results in scientific research and communication lacking a common ground. Even with the popular “V” characteristics, Big Data remains elusive. The term is broad and is used differently in research, often referring to entirely different concepts, which is rarely stated explicitly in papers. While many studies and reviews attempt to draw a comprehensive understanding of Big Data, there is a need to first retrospectively observe what Big Data actually means and refers to in concrete studies. This will help clarify ambiguities and enhance understanding of the role of Big Data in science. It will facilitate clearer communication among researchers by providing a framework for a common understanding of what Big Data entails in different contexts, which is crucial for interdisciplinary collaboration. To address this gap, we conducted a systematic literature review (SLR) of secondary studies to provide a comprehensive overview of how Big Data is used and understood across different scientific domains. Our objective was to monitor the application of the Big Data concept in science, identify which technologies are prevalent in which fields, and investigate the discrepancies between the theoretical understanding and practical usage of the term.

Our study found that various Big Data technologies are being used in different scientific fields, including machine learning algorithms, distributed computing frameworks, and other tools. These manifestations of Big Data can be classified into four major categories: abstract concepts, large datasets, machine learning techniques, and the Big Data ecosystem. All these aspects combined represent the true meaning of Big Data. This study revealed that despite the general agreement on the “V” characteristics, researchers in different scientific fields have varied implicit understandings of Big Data. These implicit understandings significantly influence the content and discussions of studies involving Big Data, although they are often not explicitly stated. We call for a clearer articulation of the meaning of Big Data in research to facilitate smoother scientific communication.

## Footnotes

^1^ https://hadoop.apache.org/

^2^ https://cassandra.apache.org/

^3^ https://www.mongodb.com/

^4^ https://hadoop.apache.org/docs/r1.2.1/hdfs_design.html

^5^ https://hbase.apache.org/

^6^ https://flume.apache.org/

^7^ https://sqoop.apache.org/

^8^ https://hive.apache.org/

^9^ https://pig.apache.org/

^10^ https://tez.apache.org/

^11^ https://flink.apache.org/

^12^ https://zookeeper.apache.org/

^13^ https://www.proxifier.com/

## Appendix

**Table A1:** Secondary studies included in our review.

**ID**	**Title**
S12	A Review of Artificial Intelligence, Big Data, and Blockchain Technology Applications in Medicine and Global Health
S15	A Review of Data Mining with Big Data toward Its Applications in the Electronics Industry
S16	A review of machine learning and big data applications in addressing ecosystem service research gaps
S19	A Review on Machine Learning, Big Data Analytics, and Design for Additive Manufacturing for Aerospace Applications
S27	A Survey on Machine Learning-Based Mobile Big Data Analysis: Challenges and Applications
S34	A systematic literature review with bibliometric analysis of big data analytics adoption from period 2014 to 2018
S47	Application of Big Data analysis in gastrointestinal research
S51	Application of Big Data and Machine Learning in Smart Grid, and Associated Security Concerns: A Review
S63	Applications of Artificial Intelligence and Big Data Analytics in m-Health: A Healthcare System Perspective
S65	Applications of Artificial Intelligence, Machine Learning, Big Data and the Internet of Things to the COVID-19 Pandemic: A Scientometric Review Using Text Mining
S66	Applications of big data analytics to control COVID-19 pandemic
S67	Applications of big data in emerging management disciplines: A literature review using text mining
S71	Big data algorithms and applications in intelligent transportation system: A review and bibliometric analysis
S74	Big Data and Business Analytics: Trends, Platforms, Success Factors and Applications
S77	Big data and IoT-based applications in smart environments: A systematic review
S86	Big Data Features, Applications, and Analytics in Cardiology-A Systematic Literature Review
S87	Big data handling mechanisms in the healthcare applications: A comprehensive and systematic literature review
S88	Big data in cybersecurity: a survey of applications and future trends
S94	Big data storage tools using NoSQL databases and their applications in various domains: a systematic review
S96	Big Data Technology in Construction Safety Management: Application Status, Trend and Challenge
S104	Cloud Computing, Big Data, and Blockchain Technology Adoption in ERP Implementation Methodology
S132	Harnessing Big Data, Smart and Digital Technologies and Artificial Intelligence for Preventing, Early Intercepting, Managing, and Treating Psoriatic Arthritis: Insights From a Systematic Review of the Literature
S133	Health Big Data Analytics: A Technology Survey
S135	How can Big Data and machine learning benefit environment and water management: a survey of methods, applications, and future directions
S161	Privacy Prevention of Big Data Applications: A Systematic Literature Review
S172	Review of Big Data and Processing Frameworks for Disaster Response Applications
S178	Review on big data applications in safety research of intelligent transportation systems and connected/automated vehicles
S181	Scoping review and bibliometric analysis of Big Data applications for Medication adherence: an explorative methodological study to enhance consistency in literature
S194	Systematic Review of the Literature on Big Data in the Transportation Domain: Concepts and Applications
S203	The Byzantine Role of Big Data Application in Nursing Science: A Systematic Review
S207	The medical and societal impact of big data analytics and artificial intelligence applications in combating pandemics: A review focused on COVID-19
S216	Use and Adaptations of Machine Learning in Big Data-Applications in Real Cases in Agriculture
S217	Use of Big Data and Information and Communications Technology in Disasters: An Integrative Review
S221	A comprehensive review of big data analytics throughout product lifecycle to support sustainable smart manufacturing: A framework, challenges and future research directions
